# Anti-inflammatory *Bifidobacterium strains* prevent dextran sodium sulfate induced colitis and associated gut microbial dysbiosis in mice

**DOI:** 10.1038/s41598-020-75702-5

**Published:** 2020-10-29

**Authors:** Shashank Singh, Ruchika Bhatia, Pragyanshu Khare, Shikha Sharma, Sivasubramanian Rajarammohan, Mahendra Bishnoi, Sanjay Kumar Bhadada, Shyam Sunder Sharma, Jaspreet Kaur, Kanthi Kiran Kondepudi

**Affiliations:** 1 Healthy Gut Research Group, Food and Nutrition Biotechnology Division, National Agri-Food Biotechnology Institute, S.A.S. Nagar, Punjab 140306 India; 2 Department of Biotechnology, University Institute of Engineering and Technology (UIET), Panjab University, Chandigarh, 160014 India; 3 Department of Endocrinology, Post Graduate Institute of Medical Education and Research (PGIMER), Chandigarh, 160012 India; 4 Department of Pharmacology and Toxicology, National Institute of Pharmaceutical Education and Research (NIPER), S.A.S. Nagar, 160062 India

**Keywords:** Dysbiosis, Ulcerative colitis

## Abstract

Crohn’s and ulcerative colitis are common inflammatory conditions associated with Inflammatory bowel disease. Owing to the importance of diet based approaches for the prevention of inflammatory gut conditions, the present study was aimed to screen the human isolates of *Bifidobacterium* strains based on their ability to reduce LPS-induced inflammation in murine macrophage (RAW 264.7) cells and to evaluate prioritized strains for their preventive efficacy against ulcerative colitis in mice. Twelve out of 25 isolated strains reduced the production of LPS-induced nitric oxide and inflammatory cytokines. Furthermore, three strains, *B. longum* Bif10*, B. breve* Bif11*, and B. longum* Bif16 conferred protection against dextran sodium sulfate induced colitis in mice. The three strains prevented shortening of colon, spleen weight, percentage body weight change and disease activity index relative to colitis mice. Lower levels of Lipocalin-2, TNF-α, IL-1β and IL-6 and improved SCFA levels were observed in *Bifidobacterium* supplemented mice relative to DSS counterparts. Bacterial composition of *B. longum* Bif10 and *B. breve* Bif11 fed mice was partly similar to the normal mice, while DSS and *B. longum* Bif16 supplemented mice showed deleterious alterations. At the genus level, *Bifidobacterium* supplementation inhibited the abundances of pathobionts such as *Haemophilus*, *Klebsiella* and *Lachnospira* there by conferring protection.

## Introduction

Crohn’s disease (CD) and Ulcerative colitis (UC) are the inflammatory gut disorders classified under Inflammatory Bowel Disease (IBD). Symptoms of IBD include chronic abdominal pain, bloating, diarrhea, blood in the stool, and inflammation of the gastrointestinal tract (GIT) and symptoms may vary among individuals based on disease location, activity and behavior^1^. The rapid rise in the prevalence of IBD across all genders, socioeconomic groups and ethnicities, makes it a burden all over the world including India^2^. India has highest disease burden and rate of incidence of UC in the Asian continent^3^. Besides physiological and genetic factors, environmental and psychological changes like stress and lifestyle can lead to the onset of IBD^4^. Response to the antibiotic-based treatments of IBD suggests the involvement of intestinal bacteria in the etiology of disease^5^. Bacteria and their components like lipopolysaccharide (LPS) leaks through the intestinal wall and translocates into the peripheral organs through disintegrated gut walls. Higher systemic level of LPS are strongly associated with enhanced inflammation, mucus depletion, increased gut permeability, and that might be responsible for IBD^6^. Healthy individuals with functional variants in *NOD2*, *IRGM*, *ATG16L1*, *CARD9* and *FUT2* genes involved in bacterial handling had decreased acetate to butyrate converting *Roseburia* spp., are at high risk for IBD^7^. Similarly, muramyl dipeptide (MDP), from Gram positive and negative bacteria, coded through NOD2 is involved in immune responses towards IBD^8^. Drugs like 5-amino salicylic acid (sulfasalazine and mesalamine), corticosteroids (hydrocortisone or methyl prednisolone) and immuno-modulating drugs Azathioprine and methotrexate are widely used, but have side-effects such as decreased white blood cells, diarrhea, pancreatitis, high blood sugar levels, high blood pressure and insomnia compromising the quality of life^1,9^. Treatment with monoclonal cytokines, i.e., anti-TNF-α and IL-6, can suppress the inflammatory activity and results in remission of IBD^10^, but this could not gain popularity owing to its high cost^11^. Hence, cost effective therapeutic and preventive approaches with minimum side effects are always in demand.

Gut microbial modulation has been suggested as an effective and safer strategy to treat IBD, which can be achieved by including probiotics, prebiotics, synbiotics, cobiotics and phytochemicals such as dietary fibers and polyphenols. Microbial modulation has been shown to be advantageous in multiple immune and inflammation related disorders including obesity^12^. Among probiotic bacteria, the genera *Bifidobacterium* is an anaerobic, Gram positive, pleomorphic, and the most vital bacterial genus and is vastly recognized for its health promoting effects on the host. With ageing, the abundance of bifidobacteria declines, while its supplementation has been shown to delay ageing, prevents UC and its associated disorders in animal models^13^.

Only limited reports exist on Indian isolates of Bifidobacteria with an ability to prevent LPS-induced and DSS induced colonic inflammation. Therefore, the present study was taken up to screen the newly isolated *Bifidobacterium* strains from infant and adult faeces for their protection against LPS-induced inflammation in murine macrophages. Furthermore, based on the aforesaid property as well as probiotic attributes, *B. longum* Bif10, *B. breve* Bif11 and *B. longum* Bif16 were prioritized and evaluated for their ability to reduce DSS induced colitis in mice.

## Results

### Screening for *Bifidobacterium* strains that could reduce nitric oxide production by LPS challenged macrophage cells

Based on the ability of the strains to curtail NO production by LPS induced macrophages and lack of NO production by per se treatment of bacteria on macrophages, Bif4, 6, 10, 11, 12, 14, 16, 17, 20, 29, 30 and 40 were shortlisted for further studies (Table 1). These strains showed reduction in TNF-α, IL-1β and IL-6 production (Table 2). *B. longum* Bif10, *B. breve* Bif11, *B. longum* Bif12 and *B. longum* Bif16, by virtue of having better reduction ability of inflammatory cytokines and probiotic potentials were short listed for detailed in vitro and in vivo studies.

**Table 1:** *Bifidobacterium* strains used in the study and their effect on NO production upon co-treatment with LPS on murine macrophages.

Strain	Source	NO production (%)	
		RAW cells + Bif	RAW cells + LPS + Bif
*B. breve* Bif 1	Infant 1	58.1 ± 1.5*	59.2 ± 3.2^#^
*B. breve* Bif 2	Infant 1	45.4 ± 3.3*	58.8 ± 3.6^#^
*B. pseudocatenulatum* Bif 3	Infant 2	44.2 ± 1.3*	4.6 ± 0.6^#^
*B. pseudocatenulatum* Bif 4	Infant 2	20.9 ± 1.3*	30.7 ± 1.8^#^
*B. longum* Bif 6	Infant 3	5.8 ± 0.6	7.4 ± 1.2^#^
*B. longum* Bif 10	Infant 4	4.0 ± 0.2	9.0 ± 1.3^#^
*B. breve* Bif 11	Infant 4	11.2 ± 1.4	22.2 ± 2.3^#^
*B. longum* Bif 12	Infant 5	13.9 ± 1.5	19.0 ± 3.4^#^
*B. longum* Bif 14	Infant 5	7.2 ± 1.4	11.4 ± 2.0^#^
*B. longum* Bif 16	Infant 5	6.3 ± 0.4	15.4 ± 2.0^#^
*B. longum* Bif 17	Adult 1	6.3 ± 0.6	8.8 ± 2.2^#^
*B. longum* Bif 18	Adult 1	16.3 ± 4.6	78.8 ± 3.0^#^
*B. longum* Bif 20	Adult 1	4.6 ± 0.3	6.7 ± 0.7^#^
*B. bifidum* Bif 21	Adult 1	88.3 ± 1.1*	87.2 ± 1.1^#^
*B. bifidum* Bif 22	Adult 1	45.4 ± 1.3*	61.0 ± 2.1^#^
*B. bifidum* Bif 23	Adult 1	38.8 ± 3.2*	73.2 ± 2.1^#^
*B. bifidum* Bif 24	Adult 1	82.5 ± 1.8*	90.0 ± 2.7
*B. longum* Bif 26	Adult 1	22.7 ± 6.0*	44.6 ± 1.6^#^
*B. longum* Bif 28	Adult 1	7.5 ± 0.5*	7.0 ± 0.19^#^
*B. longum* Bif 29	Adult 1	22.4 ± 4.3*	25.1 ± 5.1^#^
*B. longum* Bif 30	Adult 2	5.7 ± 0.5	6.4 ± 0.8^#^
*B. pseudocatenulatum* Bif 34	Adult 2	21.3 ± 1.4*	37.8 ± 3.1^#^
*B. pseudocatenulatum* Bif 37	Adult 2	32.0 ± 3.2*	43.9 ± 3.7^#^
*B. pseudocatenulatum* Bif 38	Adult 2	19.6 ± 2.5*	28.9 ± 28.9^#^
*B. breve* Bif 40	VSL#3	6.4 ± 1.0	11.5 ± 4.2^#^

Nitric oxide production for untreated RAW cells was 0.85 ± 0.01 μm/ml and for LPS treated RAW cells was 2.3 ± 0.2 μm/ml.

Data was analyzed using one-way ANOVA followed by Tukey’s Post-hoc test (*p* ≤ 0.05).

^#^Significant relative to LPS treatment.

*Significant relative to untreated control; (N = 10).

**Table 2:** Effect of Bifidobacteria and LPS co-treatment on pro-inflammatory cytokine production by murine macrophages.

Strain	Pro-inflammatory cytokine production					
	TNF-α (pg/ml)		IL-1β (pg/ml)		IL-6 (ng/ml)	
	RAW 264.7 cells + Bif	RAW 264.7 cells + LPS + Bif	RAW 264.7 cells + Bif	RAW 264.7 cells + LPS + Bif	RAW 264.7 cells + Bif	RAW 264.7 cells + LPS + Bif
*B. longum* Bif 4	2115 ± 78*	2402 ± 5.9^#^	60.4 ± 2.5*	93.3 ± 6.9^#^	14.6 ± 0.9*	48.9 ± 0.1^#^
*B. longum* Bif 6	34.3 ± 9.3	47.2 ± 12^#^	22.5 ± 1.5*	18.7 ± 3.8^#^	3.3 ± 0.3	12.1 ± 0.6^#^
*B. longum* Bif 10	50 ± 11.3	50.5 ± 12^#^	19.7 ± 0.2*	21.2 ± 3.6^#^	2.7 ± 0.4	3.11 ± 0.2^#^
*B. breve* Bif 11	72 ± 12.1*	60.6 ± 19.8^#^	29.1 ± 1.7*	22.5 ± 6.3^#^	3.9 ± 1.3	42.1 ± 1.8^#^
*B. longum* Bif 12	46 ± 15.5	98.8 ± 7.3^#^	8.4 ± 2.7	9.6 ± 3.9^#^	3.2 ± 0.2	9.7 ± 0.1^#^
*B. longum* Bif 14	28.6 ± 1.3	64.8 ± 0.6^#^	12.4 ± 4.6	33.1 ± 5.2^#^	8.1 ± 0.3	16.6 ± 1.7^#^
*B. longum* Bif 16	32.1 ± 6.1	82.6 ± 9.8^#^	4.4 ± 0.7	18.8 ± 4.9^#^	4.4 ± 0.1	6.7 ± 0.4^#^
*B. longum* Bif 17	27 ± 6.0	121.5 ± 27^#^	18.7 ± 4.6	24 ± 1.16^#^	6.0 ± 0.2	18.1 ± 1.2^#^
*B. longum* Bif 20	40.4 ± 0.1	141.2 ± 51.6^#^	11.6 ± 2.5	23.4 ± 1.1^#^	9.5 ± 0.1	61.2 ± 6.8^#^
*B. longum* Bif 29	209 ± 65.4	226.8 ± 57.3^#^	19.7 ± 4.6	26.4 ± 4.5^#^	5.6 ± 0.6	19.3 ± 2.6^#^
*B. longum* Bif 30	91 ± 22.1	392 ± 48.1^#^	22.4 ± 4.8	26.4 ± 5.3^#^	5.7 ± 1.6	24.8 ± 2.4^#^
*B. breve* Bif 40	77 ± 26.5	100 ± 3.2^#^	24.1 ± 0.4	19.7 ± 3.3^#^	4.0 ± 0.3	11.4 ± 1.6^#^

Control was untreated RAW cells; LPS control was RAW cells treated with 1 μg/ml LPS. TNF-α, IL-1β and IL-6 for untreated RAW cells were 48.5 ± 14.7 pg/ml; 3.0 ± 0.1 pg/ml and 4.9 ± 3.1 ng/ml respectively while that of LPS treated RAW cells were 4573 ± 58.8 pg/ml; 170 ± 4.5 pg/ml; 166.6 ± 10.9 pg/ml respectively.

Data was analyzed using one-way ANOVA followed by Tukey’s Post-hoc test (*p* ≤ 0.05). ^#^significant relative to LPS treatment; *significant relative to untreated control (N = 4).

### Effect of various treatments on macrophage cells and determination of NO

There was no reduction in NO production when the macrophages were incubated with killed (autoclaved) *Bifidobacterium* strains and LPS. Bacterial culture supernatants obtained from the live cultures of *B. longum* Bif10, *B. breve* Bif11, *B. longum* Bif12 and *B. longum* Bif16 did not inhibit LPS induced NO production by the macrophages.

Study on adhesion of LPS to the bacteria suggested that supernatants obtained from the mixtures of live cells of *B. longum* Bif10, *B. breve* Bif11, *B. longum* Bif12 and *B. longum* Bif16 and LPS led to 27.6, 25, 25, 35% of NO production by the macrophages while the live cell pellets from the mix caused 21, 26.4, 20 and 24% of NO production respectively relative to LPS alone treated macrophages (Table 3). Supernatants obtained from the mixtures of dead *B. longum* Bif10, *B. breve* Bif11, *B. longum* Bif12 and *B. longum* Bif16 and LPS caused 75, 69, 63 and 67% of NO production while the dead cell pellets caused 43, 24, 44 and 48.8% of NO production by the macrophages relative to LPS alone treated macrophages (Table 3).

**Table 3:** Effect of supernatants obtained from live and dead bacteria and LPS mixes on nitric oxide production by the murine macrophages.

Strains	% Nitric oxide (NO) production			
	LPS + live bacteria co-incubation		LPS + dead cells co-incubation	
	RAW cells + Pellet	RAW cells + Supernatant	RAW cells + Pellet	RAW cells + Supernatant
*B. longum* Bif 10	21.3 ± 0.2^#^	27.6 ± 0.5^#^	42.9 ± 0.9^#^	74.6 ± 1.0^#^
*B. breve* Bif 11	26.4 ± 0.4^#^	25.0 ± 1.8^#^	23.9 ± 1.0^#^	69.0 ± 1.2^#^
*B. longum* Bif 12	19.8 ± 0.4^#^	25.1 ± 0.3^#^	44.3 ± 1.0^#^	62.8 ± 0.2^#^
*B. longum* Bif 16	24.0 ± 1.2^#^	34.7 ± 3.5^#^	48.8 ± 2.4^#^	67.2 ± 0.4^#^

Nitric oxide production by the RAW cells treated with LPS was 2.3 ± 0.2 μm/ml.

Data was analyzed using one-way ANOVA followed by Tukey’s Post-hoc test (*p* ≤ 0.05). ^#^significant relative to LPS treatment (N = 5).

Based on the above results, *B. longum* Bif10, *B. breve* Bif11, *B. longum* Bif12 and *B. longum* Bif16 were assessed for their protective role in DSS induced experimental colitis in Balb/c mice.

### *Bifidobacterium* strains protected mice against DSS induced colonic inflammation

**Figure 1:**
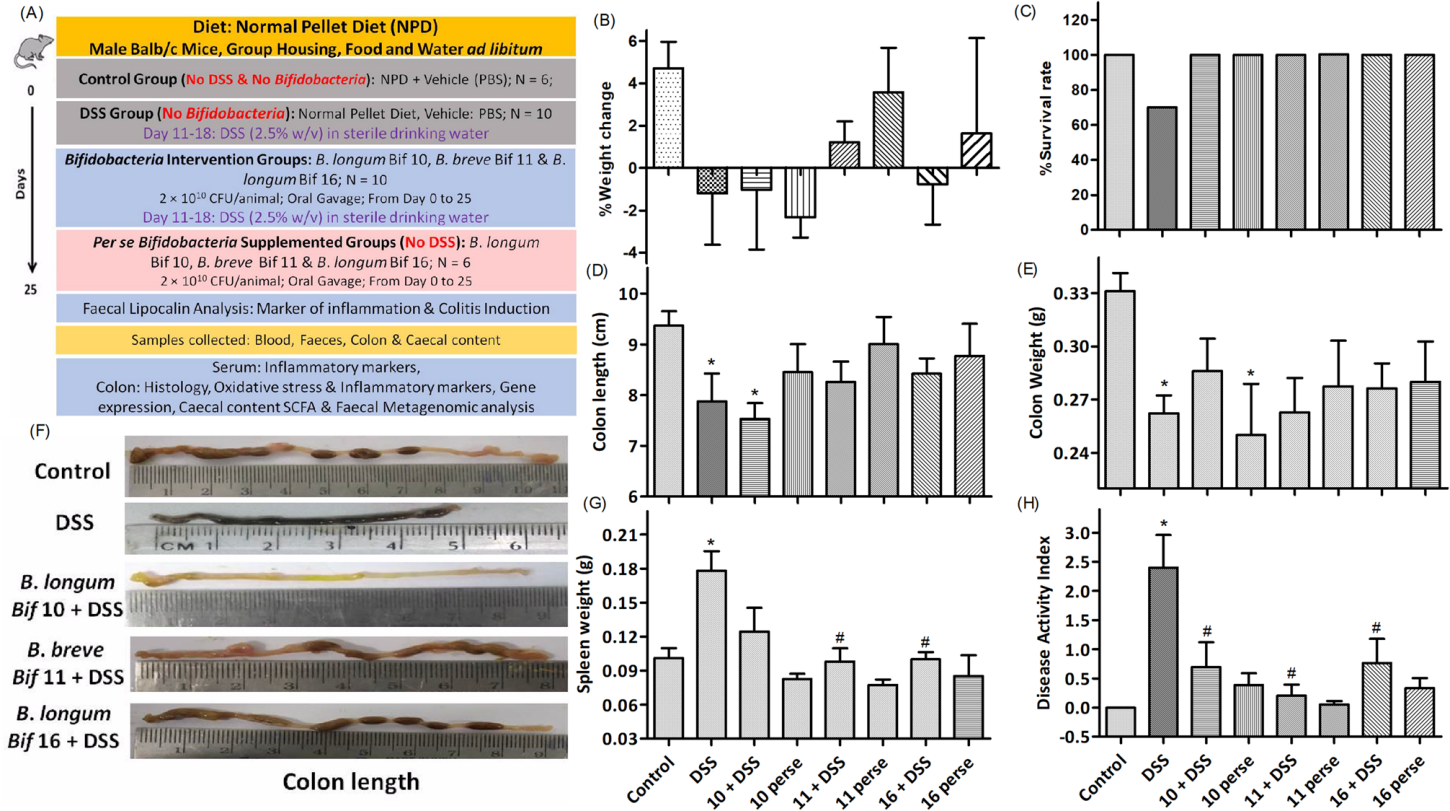
Effect of *Bifidobacterium* strains on DSS induced colitis: (**A**) Schedule of the experiment and various experimental groups; (**B**) percentage weight change; (**C**) percentage survival rate; (**D**) colon length; (**E**) colon weight; (**F**) representative colon length images; (**G**) spleen weight and (**H**) disease activity index. Data was analyzed using one-way ANOVA followed by Tukey’s Post-hoc test (*p* ≤ 0.05). *Significant relative to control; ^#^significant relative to DSS treated animals; N = 6 for control and per se groups; N = 10 for DSS and intervention groups.

DSS treatment slightly decreased the body weight of mice (Fig. 1B); caused 30% mortality (Fig. 1C), decreased the colonic length and weight (Fig. 1D,E,F), spleen weight (Fig. 1G) and promoted DAI (Fig. 1H) relative to normal mice. Mice pre-fed with *Bifidobacterium* strains prior to DSS treatment showed no effect on body weight, no effect on colon length and weight relative to normal mice (Fig. 1B,D,E). Mice in *B. breve* Bif11 and *B. longum* Bif16 groups had lower spleen weight, while that in *B. longum* Bif10 group showed a slight decrease (Fig. 1G) relative to DSS group. Mice in the *B. longum* Bif10, *B. breve* Bif11, and *B. longum* Bif16 showed lower DAI relative to DSS group (Fig. 1H).

#### *Bifidobacterium* strains protected mice from DSS induced oxidative stress in colon

**Figure 2:**
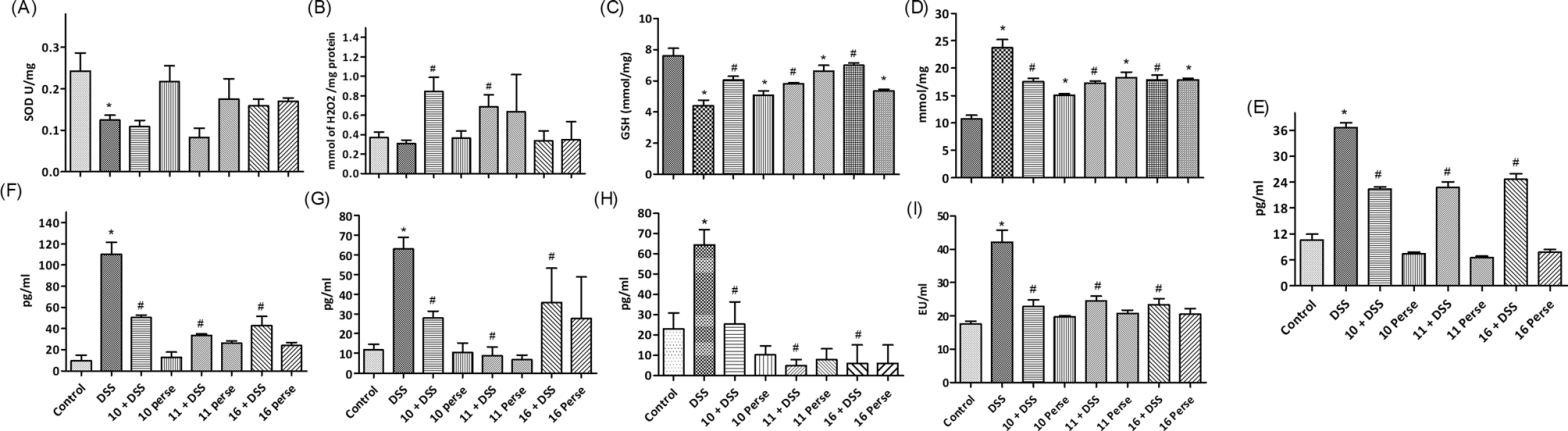
Effect of *Bifidobacterium* strains on oxidative stress and inflammatory markers: (**A**) SOD; (**B**) Catalase; (**C**) GSH; (**D**) MDA; (**E**) lipocalin-2; (**F**) TNF-α; (**G**) IL-1β; (**H**) IL-6 and (**I**) LPS. Data was analyzed using one-way ANOVA followed by Tukey’s Post-hoc test (*p* ≤ 0.05). *Significant relative to control; ^#^significant relative to DSS treated animals (N = 5).

DSS treatment decreased the SOD activity and GSH levels; increased the MDA levels, while catalase activity remains unchanged relative to normal mice (Fig. 2A–D). *B. longum* Bif10 + DSS; *B. breve* Bif11 + DSS and *B. longum* Bif16 + DSS fed mice had elevated levels of catalase and GSH, while MDA levels were reduced. SOD remained unchanged relative to DSS alone fed mice.

#### *Bifidobacterium* strains protected mice from DSS induced systemic inflammation

DSS treatment enhanced the faecal levels of lipocalin and systemic levels of TNF-α, IL-1β, IL-6 and LPS compared to normal mice, while *B. longum* Bif10 + DSS; *B. breve* Bif11 + DSS and *B. longum* Bif16 + DSS fed mice had lower levels of them relative to DSS alone fed mice (Fig. 2E–I). Importantly, per se supplementation of the strains did not promote their levels relative to normal mice.

#### *Bifidobacterium* strains modulated DSS induced deleterious gene alterations in the colon

**Figure 3:**
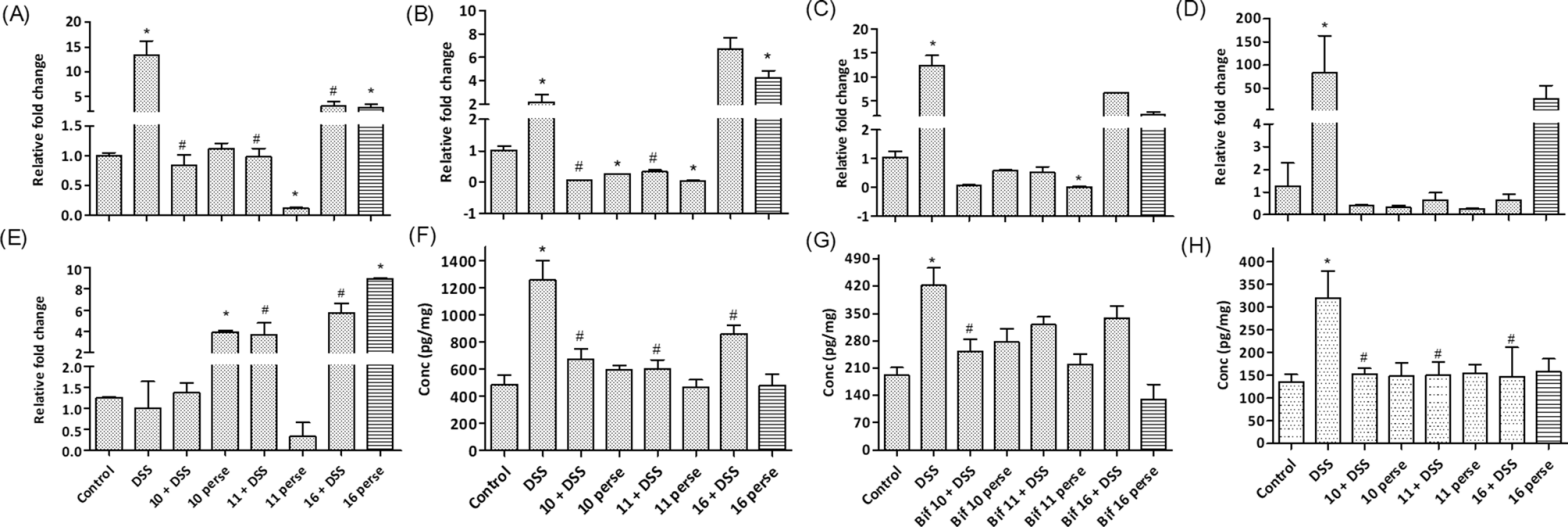
Relative expression of important genes (**A**–**E**) in the colon and (F–H) cytokines in the colon homogenates: (**A**) TNF-α; (**B**) IL-1β; (**C**) IL-6; (**D**) Ccl5; (**E**) Muc-2; (**F**) TNF-α; (**G**) IL-1β and (**H**) IL-6. Data was analyzed using one-way ANOVA followed by Tukey’s Post-hoc test (*p* ≤ 0.05). *Significant relative to control; ^#^significant relative to DSS group animals (N = 6).

DSS feeding elevated the expression of proinflammatory cytokines such as *TNF-α*, *IL-1β*, *IL-6* and *Ccl5,* while it had no effect on *MUC-2* expression relative to control mice (Fig. 3A–E). Colon homogenates showed elevated protein levels of TNF-α, IL-1β and IL-6 in the DSS treated mice (Fig. 3F–H). *B. longum* Bif10 + DSS; *B. breve* Bif11 + DSS and *B. longum* Bif16 + DSS lowered the levels of TNF-α and IL-6, whereas *B. longum* Bif10 fed mice also showed a reduction in IL-1β. Per se*, B. longum* Bif10, *B. breve* Bif11 and *B. longum* Bif16 fed mice had levels of these markers similar to normal mice.

#### *Bifidobacterium* supplementation beneficially modulated the gut microbiota

**Figure 4:**
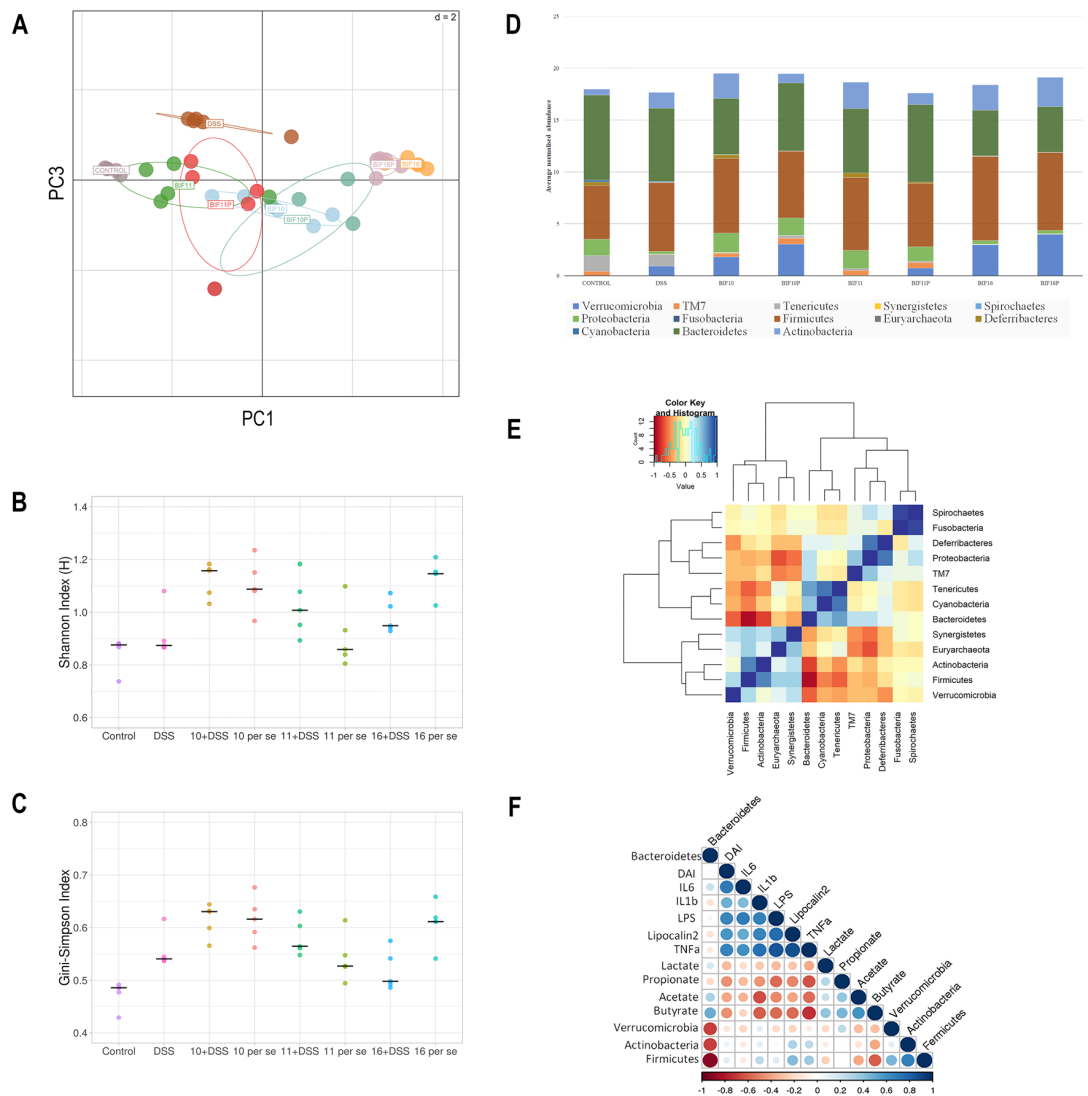
Effect of *Bifidobacterium* supplementation on gut microbial changes in DSS induced colitis in mice: (**A**) PCA analysis; (**B**) Shannon index; (**C**) Gini-Simpson index; (**D**) Bacterial abundance at phylum level; (**E**) Correlation analysis at phylum level and (**F**) Correlation analysis among different parameters. Data was analyzed using one-way ANOVA followed by Tukey’s Post-hoc test (*p* ≤ 0.05) for Shannon index, Gini-Simpson index and Bacterial abundance at phylum level. *Significant relative to control; ^#^significant relative to DSS group animals (N = 5).

Illumina paired-end sequencing of the V3-V4 region of the *16S rRNA* of the faecal samples suggested that the gut microbiota of the DSS treated mice was different from that of the mice in the control group as determined by PCA (Fig. 4A). *B. longum* Bif10 + DSS and *B. breve* Bif11 + DSS supplemented mice groups clustered together and were partly close to the control group indicating that *B. longum* Bif10 and *B. breve* Bif11 supplementation could alleviate the metagenomic changes caused by DSS challenge. However, *B. longum* Bif16 + DSS supplemented mice clustered separately, indicating a very distinct microbial diversity than rest of the groups. Species richness, as determined by the Shannon index and the evenness measure by Gini-Simpson index were almost similar in all the groups (Fig. 4B, C). At the phylum level, a total of 13 phyla were identified in all the samples. The DSS supplementation shifted the microbiome towards higher abundance of Actinobacteria (*p* = 0.004), Firmicutes (*p* = 0.003) and Verrucomicrobia and lower abundance of Bacteroidetes as compared to normal mice (Fig. 4D). *B. breve* Bif11 + DSS and *B. longum* Bif16 + DSS feeding increased the abundance of Actinobacteria in the DSS supplemented mice (*p* = 0.01 and *p* = 0.005 respectively). The relative abundance of Defferibacteres and Proteobacteria in the *B. longum* Bif10 + DSS and *B. breve* Bif11 + DSS supplemented groups was decreased as compared to the DSS fed group and was comparable to the control group (Fig. 4D). The relative abundance of Verrucomicrobia in the *B. breve* Bif11 + DSS supplemented group was decreased as compared to the DSS treated group and comparable to the control group. However, the relative abundances of Verrucomicrobia and Firmicutes were increased in the *B. longum* Bif16 + DSS supplemented group as compared to both the DSS fed and control groups (Fig. 4D).

Specifically, our results suggested that the *B. breve* Bif11 + DSS supplemented group followed by *B. longum* Bif10 + DSS group had the microbiota partly close to the normal mice, which otherwise perturbed by DSS treatment.

At the genus level, DSS treated mice had higher abundances of *Klebsiella*, *Haemophilus* and *Lachnospira* (*p* < 0.05) relative to the control mice. *B. longum* Bif10 + DSS, *B. breve* Bif11 + DSS supplemented mice showed lower abundance of *Klebsiella*, *Haemophilus* and *Lachnospira* relative to DSS treated mice while the abundance of these bacteria in the *B. longum* Bif16 + DSS group were similar to that of the DSS group. Per se supplementation of *B. longum* Bif10 and *B. breve* Bif11 did not increase their abundances relative to control, while *B. longum* Bif16 supplementation increased their abundance to the levels of DSS group (Fig. S1).

Co-occurring network between different phyla suggested that in the presence of Actinobacteria, the abundance of Firmicutes, Euryarchaeota and Synergistetes was enhanced, while the abundance of Bacteroidetes, Cyanobacteria, Tenericutes and Proteobacteria was decreased (Fig. 4E). Similarly, presence of Bacteroidetes results in higher abundance of Cyanobacteria, Tenericutes, TM7 and Proteobacteria, whereas Verrucomicrobia, Firmicutes, Actinobacteria and Synergistetes were decreased. Higher DAI and fecal lipocalin-2 levels were positively correlated with inflammatory markers (TNF-α, IL-1β, IL-6 and LPS) (Fig. 4F). Furthermore, inflammatory markers were inversely related to SCFA (acetate, propionate, lactate and butyrate). Bacteroidetes was positively correlated with lactate, acetate, butyrate while negatively related with propionate. Verrucomicrobia, Actinobacteria and Firmicutes were inversely related with butyrate, acetate and lactate.

#### *Bifidobacterium* strains prevented DSS induced damage to colonic architecture

**Figure 5:**
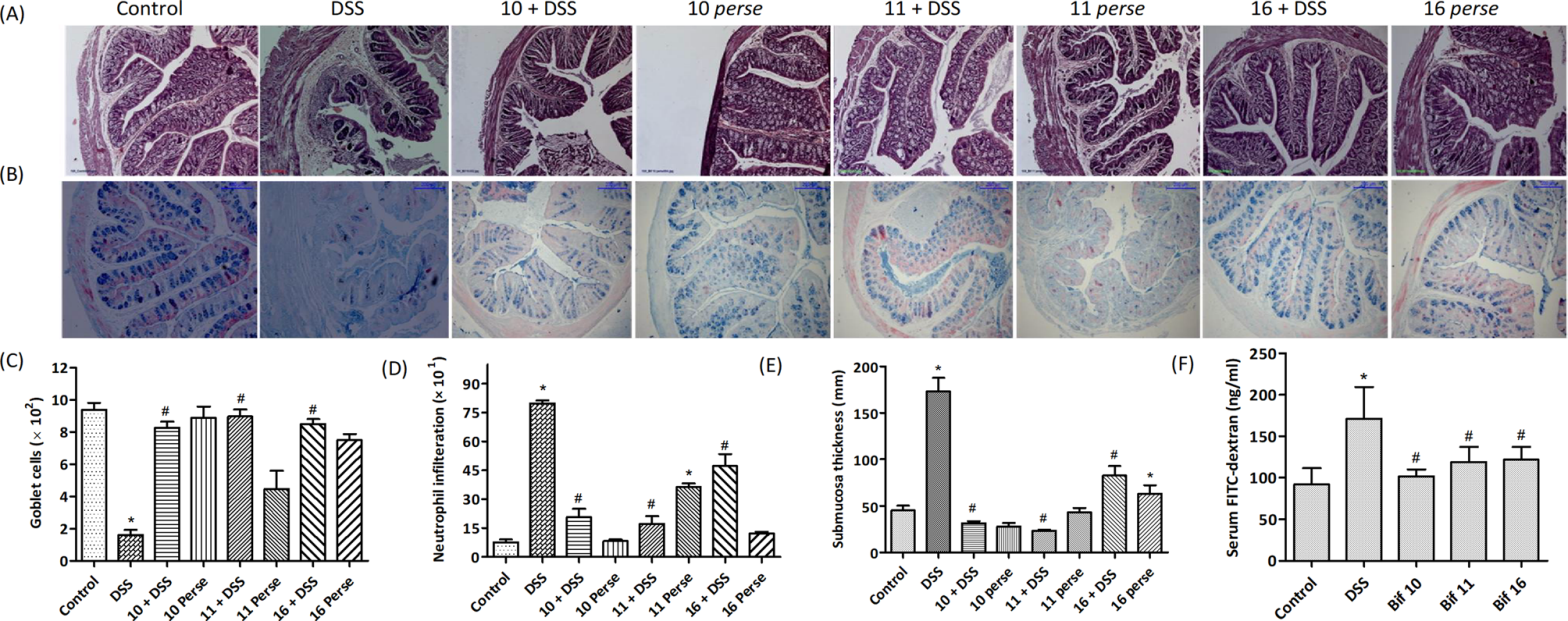
Colon histology and evaluation of gut permeability: (**A**) H&E staining; (**B**) alcian blue staining; (**C**) goblet cells number; (**D**) neutrophils infiltration; (**E**) submucosal thickness and (**F**) serum FITC-dextran. Data was analyzed using one-way ANOVA followed by Tukey’s Post-hoc test (*p* ≤ 0.05). *Significant relative to control; ^#^significant relative to DSS group (N = 4).

Histological analysis of the colon suggested that DSS feeding affected the colonic integrity and architecture along with disruption of crypt structure (Fig. 5A–E). Furthermore, the epithelial layer showed derangements with decreased number of goblet cells and higher number of infiltrated neutrophils upon DSS feeding. Sub-mucosal thickness was increased in DSS fed mice relative to normal mice. Supplementation with *B. longum* Bif10, *B. breve* Bif11 and *B. longum* Bif16 strains before DSS treatment prevented these harmful changes in the colon and conferred protection against colitis.

##### *Bifidobacterium* supplementation improved the intestinal barrier function

DSS treated mice had higher levels of FITC-dextran in the serum relative to normal mice, whereas mice supplemented with *B. longum* Bif10, *B. breve* Bif11 and *B. longum* Bif16 along with DSS had lower levels relative to DSS treated mice (Fig. 5F).

##### *Bifidobacterium* supplementation modulates the SCFA production

**Figure 6:**
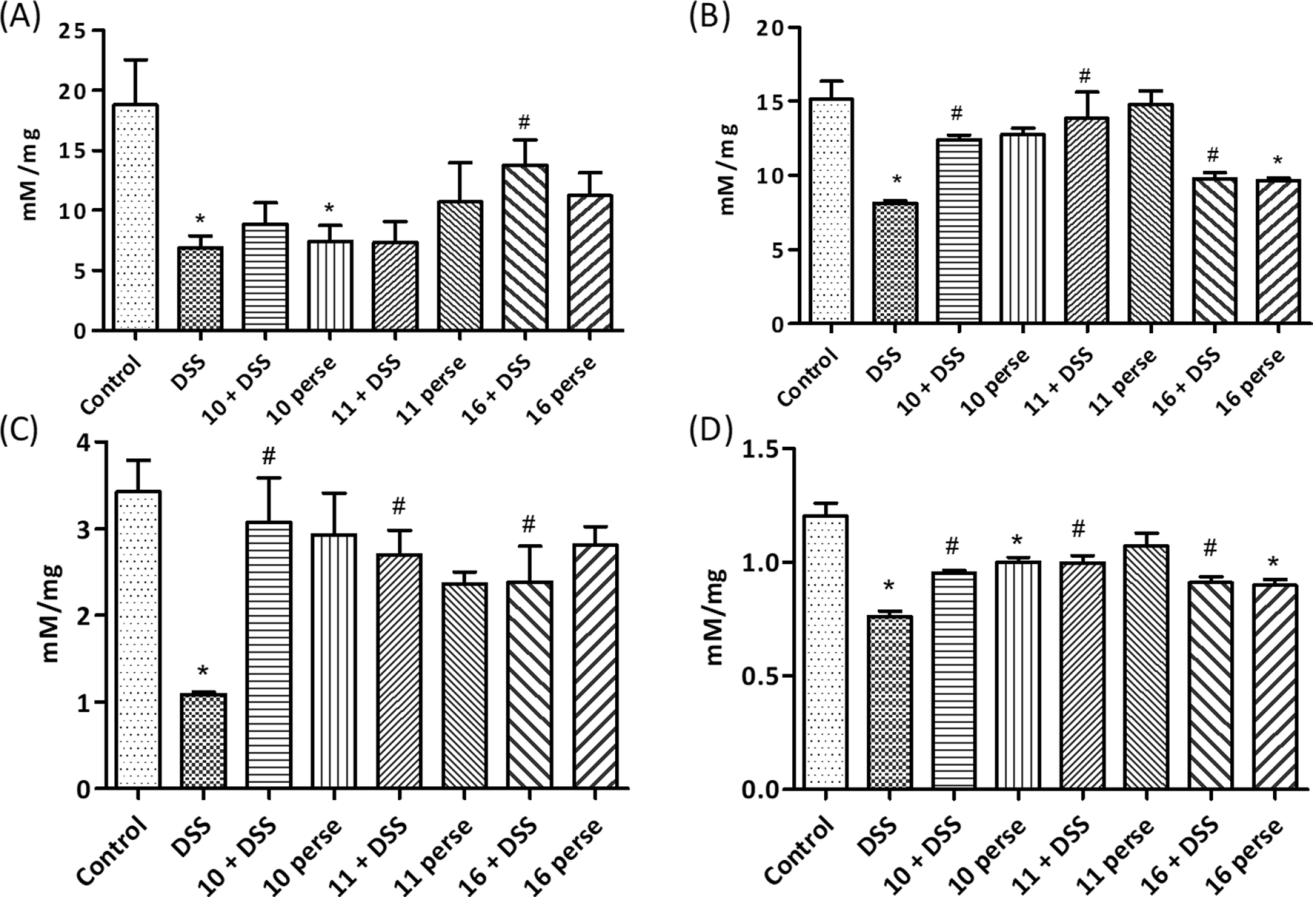
Effect of *Bifidobacterium* supplementation on the production of SCFA in caecum content in DSS induced colitis in mice: (**A**) Lactic acid; (**B**) acetic acid; (**C**) propionic acid and (**D**) butyric acid. Data was analyzed by one-way ANOVA with Tukey’s Post-hoc test (*p* ≤ 0.05). *Significant relative to control; ^#^significant relative to DSS group (N = 5).

DSS treated mice had lower lactate, acetate, propionate and butyrate levels in the caecal content relative to the control mice (Fig. 6). Lactate, acetate, propionate and butyrate were improved in the caecum of mice supplemented with *B. longum* Bif10, *B. breve* Bif11 and *B. longum* Bif16 strains (Fig. 6A–D). Lactate levels were higher in the mice pre-treated with Bif16 (Fig. 6A). Per se treatment with *B. longum* Bif16 showed decreased levels of lactate, acetate and butyrate while that of propionate remained almost the same relative to the normal mice. Per se supplementation with *B. longum* Bif10 and *B. breve* Bif11 did not affect the SCFA levels relative to normal mice, while lactate level was found to be low (Fig. 6A–D).

## Discussion

Many studies suggested that exogenous administration of *Bifidobacterium* strains improves the health, mainly due to improved barrier function, mucus production and immuno modulation besides the production of antimicrobial peptides, bacteriocins and bioactive exopolysaccharides^14,15^. Some of the in-vitro and in-vivo studies emphasized the anti-inflammatory potential of *Bifidobacterium* strains^16^. However, reports on anti-inflammatory potential of *Bifidobacterium* strains of Indian origin are scarce. In the present study, based on the ability of bacterial strains (a) to reduce nitric oxide production when treated with LPS on macrophages and (b) no stimulation of nitric oxide production when macrophages treated with *Bifidobacterium* strains alone without LPS treatment, 12 strains were shortlisted out of 25. Medina et al. suggested a lower production or no production of proinflammatory cytokines (TNF‐α and MCP-1) by the immune cells when challenged with *B. pseudocatenulatum* CECT 7765 and *B. longum* BB536 and this is a desirable feature to be considered while selecting putative probiotic strains^17^. Similarly, Okada et al. suggested that *Bifidobacterium* species combined with LPS decreased the expression levels of *IL12p40*, *IL-1β* and *TNF-α* by the macrophages in a strain dependent manner and resulted from inhibition of IκB-α phosphorylation and stimulation of SOCS signaling^18^. Our results are in congruence with the aforesaid studies and led to the identification of putative probiotic strains that do not exert pro‐inflammatory effect per se on the immune cells.

Furthermore, based on the suppression of TNFα, IL1β and IL-6 production, and probiotic attributes such as high tolerance towards acid and bile, binding to epithelial cells, binding to mucin and utilization of prebiotic isomaltooligosaccharides (unpublished data), four strains namely, *B. longum* Bif10, *B. breve* Bif11, *B. longum* Bif12 and *B. longum* Bif16 were prioritized.

Bacterial adhesion to LPS study indicated that live cells are necessary for efficient binding and elimination of LPS and thereby preventing it from binding to the Toll receptors, thus mitigating the inflammation. We have observed that supernatants from live cells + LPS mix could induce low level of NO by the macrophages, which could be due to the surface proteins on live bifidobacterial cells that could bind and eliminate LPS as suggested by Kondo et al. in *Pediococcus pentosaceous* AK-23 strain where a 217 kDa protein could bind and eliminate 38% of LPS^19^.

As *B. longum* Bif12 and *B. longum* Bif16 were isolated from the same stool sample and likely to be the same strains and since *B. longum* Bif16 showed better reduction in LPS induced IL-6 in addition to TNFα and IL1β, this was preferred over *B. longum* Bif12. Hence, three strains namely, *B*. *longum* Bif10, *B*. *breve* Bif11 and *B*. *longum* Bif16 were further evaluated in the in vivo studies.

Uncontrolled aggravation of intestinal epithelial cells causes mucosal disturbance and ulceration leading to IBD. In this study, clinical conditions of UC were reproducibly induced with 2.5% (W/V) DSS administration in drinking water for seven days, which is due to damage caused to the epithelial monolayer^20^. Supplementing *B. longum* Bif10, *B. breve* Bif11 and *B. longum* Bif16 strains to the mice protected them from colitis by alleviating crypt distortion, diarrhea, blood in the stool, reduction in colon length, neutrophil infiltration and overall improved survival rate of mice. DAI is also improved in mice fed with these strains. Our results are in alignment with that of earlier researchers where conjugated linoleic acid (CLA) producing *B. breve* and ropy EPS producing *B. longum* treatment protected the mice from DSS induced colitis^21^.

Elevated oxidative stress, due to an imbalance between ROS generation and anti-oxidant defense system, is one of the important events in acute colitis^22^. In this study, DSS treated mice had lower levels of SOD, catalase and GSH, while bifidobacteria pre-supplemented mice had higher levels of GSH and reduced levels of lipid peroxides suggesting protection from DSS associated oxidative stress induced intestinal mucosal injury. The intestinal epithelial cells are the first line of defense in any GIT disorders^23^. When the intestinal epithelial permeability dampens, invasion of bacterial associated pathogenic molecules such as LPS triggers an inflammatory response where antigen-presenting cells transform effector T-cells into Th1, Th2, Th17 and natural killer T-cells. This activates macrophages to produce TNF-α, IL-6, IL-1β and several other cytokines^24^. In our study, *Bifidobacterium* pre-feeding curtailed TNF-α, IL-6, IL-1β and LPS levels in the mice. High levels of fecal Lipocalin-2, a marker for colonic inflammation was also low in *Bifidobacterium* treated groups, suggesting protection from DSS-induced inflammation^25^.

Gene expression and protein levels of TNF-α, IL-6 and IL-1β were deleteriously altered in the colon of the DSS fed mice, as corroborated with earlier studies^21^. Supplementation with the three strains prevented the higher expression of these pro-inflammatory markers and neutrophil infiltration marker *Ccl-5* relative to DSS treated mice. *Ccl-5* is highly expressed in bacterial and viral infections and it recruits neutrophils to the site of inflammation^26^. *B. longum* Bif10 and *B. breve* Bif11 are more effective in preventing these harmful changes than *B. longum* Bif16. The mucous layer comprises of mucin and trefoil factor that obstructs the pathogen colonization in the gut. Hence, genes associated to mucus production play a critical role in gut associated infections. In this study, higher *Muc-2* mRNA expression and higher number of goblet cells in the colon suggest that *Bifidobacterium* supplementation prevented mucosal damage, which otherwise was perturbed in colitis mice.

Harmonious relationship between the gut microbiota and the host is essential for healthy living. Dysbiosis in microbiota composition leads to aberrant mucosal immunity in the gut as observed in IBD patients^27^. We have observed dysbiosis in the gut bacteria of DSS treated mice, where at the phylum level the abundance of Bacteroidetes and Deferribacters was decreased with DSS treatment, which was similar to the observation by other researchers^28^. Actinobacteria levels were low in control than DSS treated mice but increased abundance was found in interventions with *B. longum* Bif10, *B. longum* Bif11 and *B. longum* Bif16 to the mice, suggesting gut modulating effects. Similar results were shown by Cao et al.^29^. The increase in actinobacteria abundance in the intervention groups might be due to exogenously administered *Bifidobacterium* strains in the respective groups. At the genus level, *Klebsiella* abundance was increased in DSS treated animals as reported by Zhang et al.^30^. *Ruminococcus* was found in the gut of IBD patients indicating its role in the onset of colitis^31^. In our study too, increased abundance of *Ruminococcus* was observed in DSS treated mice. Members of *Blautia*, *Methanobrevebacter*, *Odoribacter* and *Lactobacillus* are the key bacteria involved in SCFA production in large intestine^30,32^. These bacteria are found in higher abundance in *Bifidobacterium* supplemented mice in this study. PCA, Shannon and Simpson indices suggested the diversity in species and its richness in gut flora between control and DSS as well as in other *Bifidobacterium* treated mice. Co-occurring pattern analysis at the phylum level suggested that *Actinobacteria* abundance was positively correlated with *Firmicutes* and *Synergistetes*. Over all, alterations in gut microbiota in DSS mice might have contributed to colitis, which is counteracted by supplementation with Bifidobacteria strains.

The protective role of SCFA against colonic inflammation has been greatly appreciated by many researchers^33^. SCFAs enhances the gut barrier function and decrease tumor formation in the colon and hence promote colonic health^34^. In the present study, DSS reduced the levels of lactate, acetate, propionate and butyrate while the three *Bifidobacterial* strains are able to prevent the drop in their levels in the caecum content relative to DSS fed mice. An oral administration of mixture of acetate (67.5 mM), butyrate (40 mM) and propionate (25.9 mM) prevented tumor development and attenuated the colonic inflammation in a mouse model of Azoxymethane (AOM)/Dextran Sodium Sulfate (DSS)-induced colitis-associated colorectal cancer^35^. Oral acetate administration has been shown to reduce inflammation in high sugar diet induced colitis in mice^36^. Supplementation with *Bacillus coagulans* MTCC5856 spores and prebiotic whole plant sugar cane fiber decreased the colitis by enhancing intestinal barrier function, reduction in oxidative stress, MPO production and decreased inflammation which in part was mediated through production of propionate and butyrate^37^. Sodium propionate administration through oral route reduced the symptoms of DSS-induced colitis by promoting intestinal barrier function and suppressing inflammation and oxidative stress through STAT3 pathway^36^. Butyrate is known to protect the intestinal tissues by promoting mucus production, decreasing inflammation, oxidative stress and exert epigenetic regulation on colonocytes^38^. The benefits of oral sodium butyrate in drinking water against TNBS induced colitis in mice suggested that butyrate enhanced the intestinal barrier function due to mucin production and reduction in inflammation^39^ and in DSS induced colitis, it increased the expression of *MUC2, MUC1*, *MUC3*, and *MUC4*^40^. However, it is desirable to manipulate the levels of SCFA within the host rather than exogenous administration, which may be challenging for chronic usage and in this context the three strains identified through this study might be useful in modulating the SCFA levels for maintaining healthy intestinal milieu and immunomodulation.

Although, the three strains protected the mice from inflammation, *B. longum* Bif 16 showed slightly lesser effects in relative mRNA levels of TNFα, IL1β and IL-6 as well as having higher abundances of *Haemophilus*, *Klebsiella* and *Lachnospira*.

**Figure 7:**
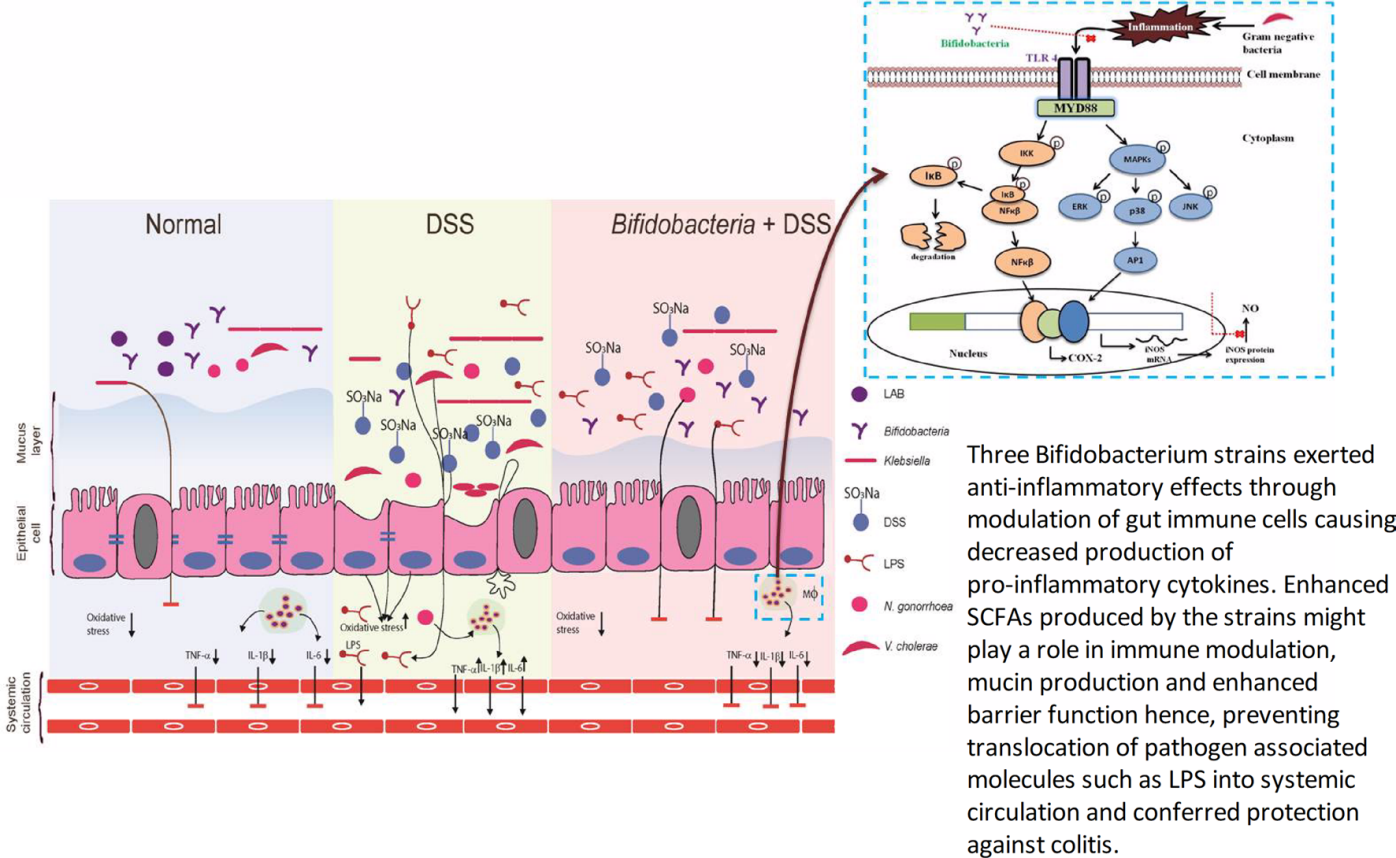
Proposed mode of action of *B. longum* Bif10 and *B. breve* Bif11 in protecting against DSS induced ulcerative colitis in mice.

In conclusion, this study suggested the protective efficacy of *B. longum* Bif10 and *B. breve* Bif11 strains against DSS induced colonic inflammation, which might be due to the direct interaction of the strains with immune cells as demonstrated through in vitro studies as well as through SCFA production that interacts with immune cells and epithelial cells as evidenced in in vivo studies (Fig. 7). SCFAs produced might have contributed to immune modulation, mucin production and enhanced barrier function and by preventing translocation of the pathogen associated molecule, LPS, into the systemic circulation. Furthermore, the protective and therapeutic efficacy of these strains in preclinical models are ongoing.

## Methods

### Bacterial strains and culture conditions

Twenty-five *Bifidobacterium* strains isolated from fresh feces of human infants and adults on modified MRS agar containing 0.05% (w/v) each of L-cysteine hydrochloride and mupirocin were used in this study. This study was approved by institute Ethical committee of Post Graduate Institute of Medical Education and Research (PGIMER), Chandigarh (ethical approval number: P-641) and all protocols of experimentation and methods were performed in accordance with the relevant guidelines and regulations. Also, informed consent was obtained from both adults and parents of the infants. Active cultures of the strains were prepared from Trypticase soy broth glycerol stocks on MRSC plates and incubated at 37 °C for 48 h under anaerobic conditions. A single colony of each strain from the agar was sub-cultured three times consecutively in fresh MRSC broth at 37 °C for 24 h. Later, the cultures were centrifuged at 8000 rpm for 10 min at 4 °C and the pellets obtained were washed and resuspended in sterile PBSC. *B. breve* Bif40, isolated from VSL#3 (De Simone Formulation, manufactured by Sigma-Tau Pharmaceuticals and marketed by Sun pharma) was used as a reference strain in all the in-vitro experiments.

### Screening for *Bifidobacterium* strains that could prevent LPS induced nitric oxide production by murine macrophage cells (RAW 264.7)

RAW 264.7 cell line was purchased from National Centre for Cell Science, Pune, India and cultured in DMEM containing 10% fetal bovine serum and 1% penicillin–streptomycin at 37 °C under 5% CO_2_ in a humidified incubator. The cells were passaged on every second day.

#### Treatment of macrophages with *Bifidobacteria* (Bif) and LPS

Confluent macrophage cells (1 × 10^5^ cells) were treated with 1 μg/ml LPS and each isolated strain (OD_600_ 1 unit ≈ 1 × 10^10^ CFU) for 16 h in a humidified incubator. The supernatants collected were used for the determination of nitric oxide production.

#### Viability assay

Confluent macrophages in a 96 well cell culture plate was treated with 1.0 OD_600_ of each bacterial strain and incubated at 37 °C under 5% CO_2_ in a humidified incubator. After 16 h, the supernatants were discarded and the viability of the cells was determined using MTT assay kit (Sigma-Aldrich, USA) as per the instructions.

### Effect of various treatments on macrophages and determination of NO

Confluent macrophages were treated with variously processed bacteria as described below, either in the presence or absence of LPS.

#### Live cells

Live bacterial cells of each strain (1 × 10^10^ CFU ≈ 1.0 OD_**600**_) were added to the macrophages and incubated for 16 h.

#### Dead cells

Freshly grown bacterial cultures for 24 h were autoclaved and the cells were harvested by centrifugation, added on the macrophage cells and incubated for 16 h as mentioned above.

#### Cell free bacterial culture supernatants

Supernatants from the bacterial cultures were obtained from the overnight grown cultures by centrifuging at 8000 rpm for 10 min at 4 °C, filter sterilized using 0.22 μm filters, lyophilized and 0.125, 0.250, 0.5 and 1 mg/ml in DMEM were prepared and added to the macrophages for 16 h as described above.

#### Adhesion of LPS to live and dead bacteria

This was determined by suspending fresh viable and dead bacterial cells (1.0 OD/ml) in DMEM having 1 μg/ml LPS and incubated at 37 °C under anaerobic conditions for 2 h. The contents were centrifuged and the supernatants and bacterial cells were added to the macrophages and incubated at 37 °C for 16 h under 5% CO_2_ in a humidified atmosphere.

#### NO determination

NO was determined using Greiss reagent. Briefly, 100 μl of Griess reagent (2% Sulfanilamide (w/v) in 5% phosphoric acid, 0.2% *N*-(1-Naphthyl) ethylenediamine dihydrochloride in H_2_O (w/v)) [1:1]) was added to 150 μl of the supernatants. The level of NO was determined by measuring OD_540_ using ELISA reader.

For the challenging experiments with live and dead bacteria, TNF-α, IL-6 and IL-1β concentrations in the supernatants were determined using commercially available ELISA kits as per the manufacturer’s instructions.

### Protection against DSS induced colonic inflammation in mice

Based on in vitro studies on murine macrophages, *B. longum* Bif10, *B. breve* Bif11, and *B. longum* Bif16 were selected for in vivo studies for evaluation of their protective efficacy against DSS induced colitis in Balb/c mice.

#### Animals

The animal study protocol was approved by Institute Ethical Committee (IAEC/17/57) of National Institute of Pharmaceutical Education and Research, S. A. S. Nagar and all the methods were performed in accordance with the relevant guidelines and regulations as per the Committee for the Purpose of Control and Supervision of Experiments on Animals guidelines (CPCSEA) for experimentation on animals, Govt. of India. Male Balb/c mice aged 8–10-week old were acclimatized for a week under standard experimental conditions at the Central Animal Facility of NIPER,  S. A. S. Nagar. Water and food were provided *ad-libitum*.

#### Experimental groups and treatment schedule

The duration of the experiment was 25 days and the schedule is given in Fig. 1A. Briefly, weight normalized mice were randomized into 8 groups as (1) Control or vehicle group (n = 6), mice were fed with normal pellet diet and gavaged with 200 μl of PBSC (vehicle); (2) DSS group (n = 10), mice were *ad-libitum* provided with 2.5% w/v of DSS in sterile drinking water; (3) *B. longum* Bif10 + DSS group (n = 10), mice were gavaged with *B. longum* Bif10 and DSS; (4) *B. breve* Bif11 + DSS group (n = 10), mice were gavaged with *B. breve* Bif11; (5) *B. longum* Bif16 + DSS group (n = 10), mice were gavaged with *B. longum* Bif16; (6) *B. longum* Bif10 per se group (n = 6), mice were gavaged with *B. longum* Bif10; (7) *B. breve* Bif11 per se group (n = 6), mice were gavaged with *B. longum* Bif11; (8) *B. longum* Bif16 per se group (n = 6), mice were gavaged with *B. longum* Bif16.

#### Induction of colitis and its prevention using *Bifidobacterium* strains

Colitis in the DSS group was induced by providing DSS (2.5% w/v) in sterile drinking water from the 11th to 18th day. From 19th day onwards, the mice were shifted to normal drinking water till the end of experiment.

#### Bacterial dose preparation and supplementation

Overnight grown bacterial cultures were centrifuged at 8000 rpm for 10 min at 4 °C and resuspended in PBSC after washing. A dose of 2 × 10^10^ CFU/ml of bacteria was prepared in trypticase soy broth (TSB) with 15% glycerol and stored at − 80 °C. At the time of dosing, stored bacterial stocks were centrifuged and suspended in approximately 250 µl of PBSC and was gavaged to each mouse on every day from the beginning till the end of the experiment while DSS was provided *ad-libitum* from 11 to 18th day as per the schedule. Body weights of the animals and survival rate were monitored. Change in body weight was determined using the below formula

$$\% \;{\text{body}}\;{\text{weight}}\;{\text{change}} = \left[ {\left( {{\text{weight D}}_{{\text{s}}} {-}{\text{weight D}}_{0} /{\text{weight D}}_{0} } \right) \times {1}00} \right]$$

where D_s_ was body weight at a specific day and D_0_ was body weight at day 0.

Induction of inflammation in various groups was determined by quantifying fecal lipocalin-2 using ELISA kit as per the manufacturer instructions. A Disease activity index (DAI) was calculated using the data on body weight loss (0, < 1%; 1, 1–5%; 2, 5–10%; 3, 10–20%; 4, > 20%), stool consistency (0, normal; 2, loose; 4, diarrhea) and gross bleeding (0, normal color; 2, reddish color; 4, bloody stool) divided by 3 for each mouse^41^.

#### Evaluation of intestinal permeability

This was evaluated as per Chassaing et al.^25^. Briefly, on the day of sacrifice (25th day), three mice from each group were starved for 4 h. FITC-dextran (0.6 mg/g body weight; 4–40 KDa, Sigma-Aldrich Co., St. Louis, MO, USA) was gavaged to each animal and after 3 h serum was collected and fluorescence was measured at 488 and 520 nm as excitation and emission wavelengths respectively. Permeability was calculated as the amount of FITC-dextran diffused into the blood circulation from the intestine relative to the normal mice.

### Biochemical analysis

Blood was collected from retro-orbital plexus of mice and serum was separated and stored at − 80 °C till further analysis. TNF-α, IL-6, IL-1β and LPS levels were measured through commercially available ELISA kits as per the manufacturer’s instructions.

At the end of the experiment, mice were sacrificed by cervical dislocation. Spleen, colon and colonic contents were harvested from each animal. Colon length, weight and spleen weight were determined.

#### Preparation of proximal colon tissue homogenate

Approximately 50 mg of proximal colon was taken to prepare 10% tissue homogenate in ice cold PBS containing protease inhibitor cocktail using a handheld homogenizer. The homogenate was centrifuged at 12,000 rpm for 15 min at 4 °C and supernatants collected were stored at -20 °C until further use. Protein content in the homogenate was determined by Bradford method. Malondialdehyde level was determined by adding 200 μl of tissue homogenate to 200 μl of 8.1% SDS, 1.5 ml of 20% acetic acid (pH 3.4), 1.5 ml of thiobarbituric acid (0.8%) and 600 μl of H_2_O in a test tube and boiled at 95 °C for 1 h in a water bath. The contents were centrifuged at 5000*g* for 10 min and the absorbance of the supernatants was measured at 532 nm. The concentration of MDA was expressed as mmol MDA/mg protein of the tissue homogenate. Catalase activity was determined as per Luck^42^. Briefly, 1.95 ml of phosphate buffer (0.05 M, pH 7.0), 1 ml of hydrogen peroxide (0.019 M) and 0.05 ml of colon tissue homogenate were mixed. Absorbance at 240 nm was measured for 2 min with 30/60 s intervals. Catalase activity was expressed as μmoles of H_2_O_2_ decomposed per min per mg of protein. Superoxide dismutase was determined as per Kono^43^. Briefly, to 0.1 ml of tissue homogenate and 0.5 ml of hydroxylamine hydrochloride (20 mM, pH 6.0 adjusted with NaOH) solution, 2 ml of nitro blue tetrazolium (96 mM, dissolved in 50 mM sodium carbonate in 0.1 mM EDTA, pH 10.8) was added. The auto-oxidation of hydroxylamine was observed by measuring the absorbance at 560 nm for 2 min at 30/60 s intervals. Reduced Glutathione levels were determined as per Ellman method^44^. Briefly, an equal volumes of tissue homogenate and sulphosalicylic acid were mixed and kept on ice for 1 h, centrifuged at 4500 g for 10 min at 4 °C. To 50 μl of clear supernatant, 450 μl of 0.01 M 5,5-dithiobis-(2-nitrobenzoic acid) in 0.4% of 0.1 M phosphate buffer was added and incubated at 37 °C for 10 min. Absorbance was measured at 412 nm.

#### Gene expression analysis in colon tissue

The list of genes and primer sequences used for RT-PCR are given in Table S1. Total RNA from colon tissue (50 mg) was extracted by Trizol method and quantified on nanodrop (Thermo fisher). cDNA was prepared by reverse transcription kit (Bio Rad, USA; catalogue number 1725035) as per manufacturer instructions. The conditions for RT-PCR were 95 °C for 5 min, 95 °C for 15 s, 60 °C for 30 s, 72 °C for 30 s and 72 °C for 5 min. ∆∆ct method was used to determine the relative fold changes in the expression levels of genes using β-actin as the house keeping gene.

#### Faecal microbiota analysis

Genomic DNA was extracted from 100 mg of freshly collected faeces using QIAmp Fast DNA stool mini kit (Qiagen) as per the manufacturer’s instructions and quantified and stored at − 80 °C. The V3–V4 region of the 16S rRNA was sequenced using llumina Hiseq platform by a commercial company (NxGenBio Life Sciences, New Delhi, India). Reads obtained were quality checked using FastQC (version 0.11.5) on the basis of base quality and sequence quality score distribution. Reads with a threshold value of Q20 or more were used for the analysis. However, majority of the reads had a quality score of Q30 or more (99.9% accuracy). Paired reads were assembled using PEAR (Paired-End reAd mergeR). OTU processing and microbiome analysis were performed by QIIME 1.9.1 and normalization of OTU’s was done by Picrust 1.3.3. Furthermore, Calypso was used to perform taxonomical classification and various analyses (Diversity, PCA, abundance plot, etc.).

#### Colon histology

Colons excised from the mice (n = 3) at the time of sacrifice were stored in 10% phosphate buffered formalin. A small piece of tissue was processed in a series of ethanol and xylene and embedded in molten paraffin. Colonic sections of approximately 5 μm thickness were cut from paraffin embedded tissue blocks using a microtome (Leica, Wetzlar, Germany). Slides were stained with hematoxylin and eosin for microscopic evaluation as per standard methods. On the basis of the architecture of colonic sections, scoring was done for each slide as described elsewhere^38^. Alcian blue staining was done on separate sections to assess the number of mucus producing goblet cells.

#### Short chain fatty acid (SCFA) analysis in caecum content

HPLC method was used for the analysis as described elsewhere^45^. Briefly, 50 mg of the caecal content was extracted with acidified water (pH 1 – 2) by rigorous vortexing followed by incubation at room temperature for 10 min. Contents were centrifuged (6000 rpm, 20 min, 4 °C) and filtered using 0.2 µm nylon filters (Millipore, Millex-GN) and Isobutyric acid (5 µM) was included as an internal standard.

HPLC system (Agilent 1260 infinity series, Agilent Technology, Singapore) equipped with a controller pump (Agilent, model no. G1311C), an auto sampler unit (model no. G1329B) and a diode array detector (DAD; model no. G1315D) set at 210 nm (peak width 2.5 Hz, band width 2 nm) fitted with anion exchange column (300 × 7.7 mm; 8 µm particle size) along with a guard column made with the same material was used. The column was equilibrated and eluted with a mobile phase comprising of formic acid (0.1% v/v) in Milli-Q water (Merck Millipore, 0.22 µm filtered, resistivity 18.1 – 18.3 MΩ cm) at an isocratic flow rate of 0.6 mL/min at 60 °C for 30 min. A 20 µL of the caecal extract in five biological replicates and acetate, propionate and butyrate standards were injected and separated at 60 °C. Data acquired was processed with EZchrom elite software with the system suitability option installed. The results obtained were expressed as µM concentration of SCFAs per mg sample against the SCFA standards.

### Statistical analysis

Graph pad PRISM-5.0 (Graph pad software Inc., CA, USA) software was used for all the analysis. Values were expressed as means ± SEM. Statistical significance between the groups was analyzed One-way analysis of variance (ANOVA) followed by Tukey’s post-hoc test. *p* ≤ 0.05 was considered as significant.

## Supplementary information

Supplementary Information

## Abbreviations

DSS: Dextran sodium sulfate
Bif: *Bifidobacterium*
NO: Nitric oxide
CFU: Colony forming unit
