# Supplementary material for: Anti-inflammatory Bifidobacterium strains prevent dextran sodium sulfate induced colitis and associated gut microbial dysbiosis in mice
Source: Sci Rep. 2020 Oct 29;10:18597. doi: 10.1038/s41598-020-75702-5 (PMC7596498; doi:10.1038/s41598-020-75702-5)
Supplement: Supplementary file 1 — Supplementary Information [file 41598_2020_75702_MOESM1_ESM.doc]

**Anti-inflammatory *Bifidobacteria* prevents Dextran Sodium Sulfate induced colitis and associated gut microbial dysbiosis in mice**

**Shashank Singh^1,2^, Ruchika Bhatia^1^, Pragyanshu Khare^1^, Shikha Sharma^1^, Sivasubramanian Rajarammohan^1^, Mahendra Bishnoi^1^, Sanjay Kumar Bhadada^3^, Shyam Sunder Sharma^4^, Jaspreet Kaur^2†^, Kanthi Kiran Kondepudi^1†^**

^1^National Agri-Food Biotechnology Institute (NABI), S.A.S. Nagar-140306, Punjab, India

^2^Department of Biotechnology, University Institute of Engineering and Technology (UIET), Panjab University, Chandigarh-160014, India

^3^Department of Endocrinology, Post Graduate Institute of Medical Education and Research (PGIMER), Chandigarh-160012, India

^4^Department of Pharmacology and Toxicology, National Institute of Pharmaceutical Education and Research (NIPER), 160062, India

**^†^**Corresponding authors:

**Dr. Kanthi Kiran Kondepudi**

Scientist-E, Healthy Gut Laboratory, Food & Nutrition Biotechnology Division

National Agri-Food Biotechnology Institute, S.A.S. Nagar-140306, Punjab, India.

Phone: +91-172-5221246. Fax: +91-172-5221100

E-mail: kiran@nabi.res.in; [kanthikiran.kondepudi@gmail.com](mailto:kanthikiran.kondepudi@gmail.com)

**Dr. Jaspreet Kaur**

University Institute of Engineering and Technology, Panjab University, Chandigarh-160014,

India, Phone: +91-172-2534967. E-mail: [jaspreet_uiet@pu.ac.in](mailto:jaspreet_uiet@pu.ac.in)

**Supplementary Information (Tables)**

**Table S1: List of primers used in this study**

| **Primer name** | **Primer sequence** |
| --- | --- |
| ***UNI 27F*** | 5’ AGAGTTTGATCCTGGCTGAG 3’ |
| ***UNI 1492R*** | 5’ TAGGGYTACCTTGTTACGACTT 3’ |
| ***XFPF*** | 5’ CGGCCACGGCTGGGGCC 3’ |
| ***XFPR*** | 5’ TCCTGACGCCAGACGTGGG 3’ |
| ***Ccl5F*** | 5’ ATCATCCTCACTGCAGCCG 3’ |
| ***Ccl5R*** | 5’ TTCTCTGGGTTGGCACACAC 3’ |
| ***Muc-2F*** | 5’ GGCCTCACCACCAAGCGTCC 3’ |
| ***Muc-2R*** | 5’ TGGGCTGGCAGGTGGGTTCT 3’ |
| ***IL-1βF*** | 5’ AATACCTGTGGCCTTGGGC 3’ |
| ***IL-1βR*** | 5’ CTCTGCTTGTGAGGTGCTGA 3’ |
| ***TNF-αF*** | 5’ AAGCCTGTAGCCCACGTCGTA 3’ |
| ***TNF-αR*** | 5’ GGCACCACTAGTTGGTTGTCTTTG 3’ |
| ***IL-6F*** | 5’ GATGGATGCTACCAAACTGGA 3’ |
| ***IL-6R*** | 5’ GAGCATTGGAAATTGGGGTA 3’ |

**Table S2:** Total number of reads obtained per sample

| **Groups** | **No. of Reads** | **Seq. Length** | **GC%** |
| --- | --- | --- | --- |
| **Control1** | 233377 | 35-251 | 54 |
| **Control2** | 224697 | 35-251 | 54 |
| **Control3** | 200459 | 35-251 | 53 |
| **Control4** | 266631 | 35-251 | 53 |
| **Control5** | 288542 | 35-251 | 53 |
|  |  |  |  |
| **DSS1** | 246356 | 35-251 | 54.5 |
| **DSS2** | 233550 | 35-251 | 54.5 |
| **DSS3** | 260378 | 35-251 | 54.5 |
| **DSS4** | 291455 | 35-251 | 54.5 |
| **DSS5** | 202803 | 35-251 | 54 |
|  |  |  |  |
| **10 + DSS1** | 258496 | 35-251 | 55.5 |
| **10 + DSS2** | 209271 | 35-251 | 54.5 |
| **10 + DSS3** | 214158 | 35-251 | 55 |
| **10 + DSS4** | 256296 | 35-251 | 54.5 |
| **10 + DSS5** | 259696 | 35-251 | 54 |
|  |  |  |  |
| **10 Perse1** | 280922 | 35-251 | 54 |
| **10 Perse2** | 241539 | 35-251 | 54 |
| **10 Perse3** | 276200 | 35-251 | 53.5 |
| **10 Perse4** | 209528 | 35-251 | 53 |
| **10 Perse5** | 235341 | 35-251 | 53 |
|  |  |  |  |
| **11 + DSS1** | 259696 | 35-251 | 54 |
| **11 + DSS2** | 241040 | 35-251 | 53.5 |
| **11 + DSS3** | 201295 | 35-251 | 53.5 |
| **11 + DSS4** | 254988 | 35-251 | 53 |
| **11 + DSS5** | 232657 | 35-251 | 54.5 |
|  |  |  |  |
| **11 Perse1** | 281760 | 35-251 | 53.5 |
| **11 Perse2** | 241547 | 35-251 | 53 |
| **11 Perse3** | 232430 | 35-251 | 54 |
| **11 Perse4** | 203040 | 35-251 | 54 |
| **11 Perse5** | 243007 | 35-251 | 53 |
|  |  |  |  |
| **16 + DSS1** | 241704 | 35-251 | 56 |
| **16 + DSS2** | 248044 | 35-251 | 56.5 |
| **16 + DSS3** | 236067 | 35-251 | 56.5 |
| **Groups** | **No. of Reads** | **Seq. Length** | **GC%** |
| **16 + DSS4** | 229281 | 35-251 | 56.5 |
| **16 + DSS5** | 238664 | 35-251 | 56 |
|  |  |  |  |
| **16 Perse1** | 198484 | 35-251 | 56.5 |
| **16 Perse2** | 67423 | 35-251 | 56 |
| **16 Perse3** | 198484 | 35-251 | 56.5 |
| **16 Perse4** | 235087 | 35-251 | 56.5 |
| **16 Perse5** | 241579 | 35-251 | 56.5 |

**
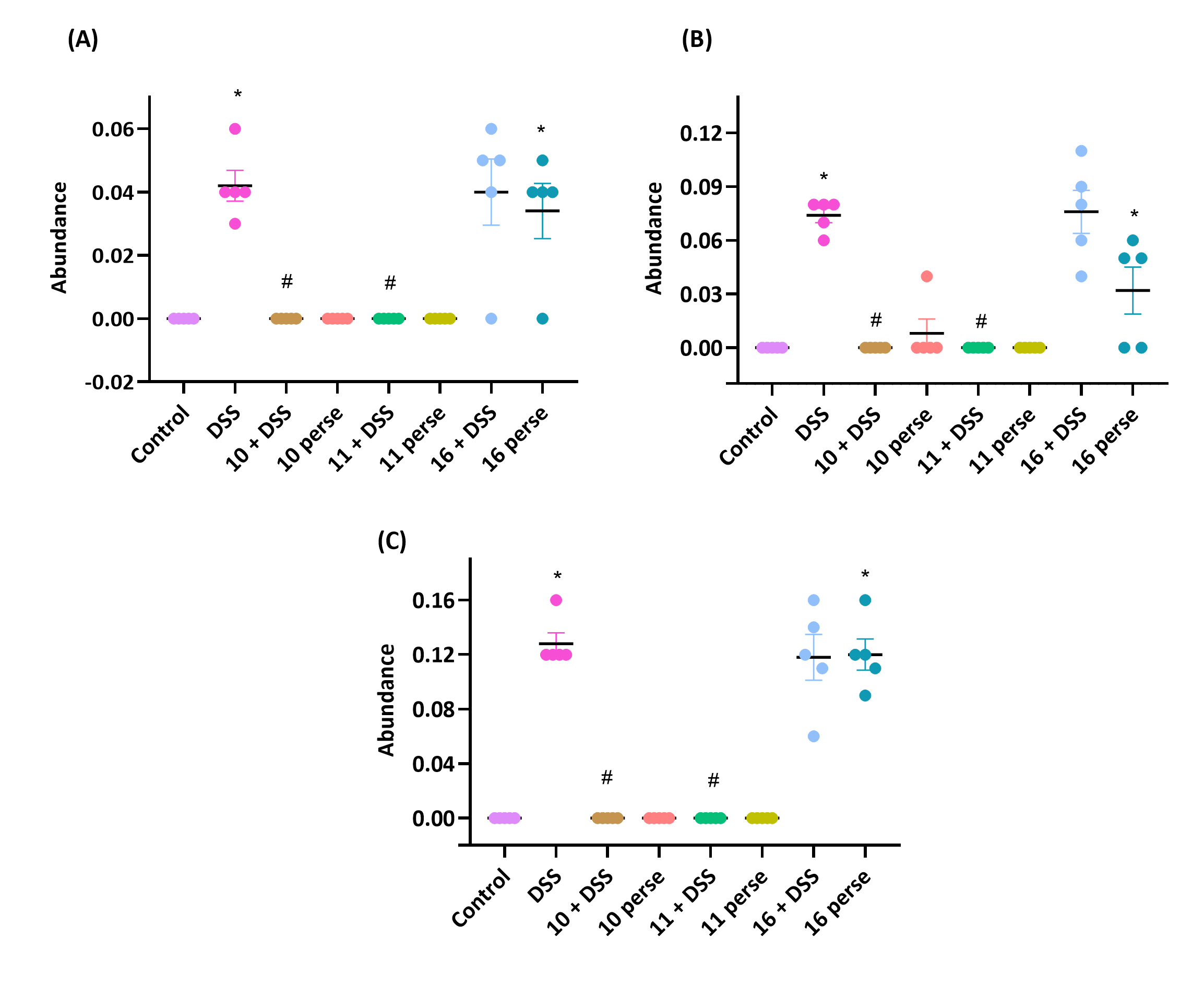
**

**Supplementary Figure S1:** Effect of *Bifidobacterium* supplementation on gut bacterial

abundance at genus level: (A) *Haemophilus*; (B) *Klebsiella* and (C) *Lachnospira*.

Data was analyzed using one-way ANOVA followed by Tukey’s Post-hoc test (P ≤ 0.05). *significant relative to control; # significant relative to DSS group (N = 5).
